# Distribution and Determinants of Plasma Homocysteine Levels in Rural Chinese Twins across the Lifespan

**DOI:** 10.3390/nu6125900

**Published:** 2014-12-18

**Authors:** Yuelong Ji, Xiangyi Kong, Guoying Wang, Xiumei Hong, Xin Xu, Zhu Chen, Tami Bartell, Xiping Xu, Genfu Tang, Fanfan Hou, Yong Huo, Xiaobin Wang, Binyan Wang

**Affiliations:** 1 Center on the Early Life Origins of Disease, Department of Population, Family and Reproductive Health, Johns Hopkins University Bloomberg School of Public Health, 615 N. Wolfe Street, E4132 Baltimore, MD 21205-2179, USA; E-Mails: yji7@jhu.edu (Y.J.); gwang24@jhu.edu (G.W.); xhong3@jhu.edu (X.H.); zchen31@jhu.edu (Z.C.); 2 Anzen Hospital, Beijing Capital Medical University, Beijing 100069, China; E-Mail: sherrykxy@gmail.com; 3 Institute of Biomedicine, Anhui Medical University, Hefei 230032, China; E-Mails: xinxxu@gmail.com (X.X.); xipingxu126@126.com (X.X.); 4 National Clinical Research Center for Kidney Disease, Nanfang Hospital, Southern Medical University, Guangzhou 510515, China; E-Mail: ffhouguangzhou@163.com; 5 Mary Ann & J. Milburn Smith Child Health Research Program, Stanley Manne Children’s Research Institute, Chicago, IL 60614, USA; E-Mail: TBartell@luriechildrens.org; 6 School of Health Administration, Anhui Medical University, Hefei 230032, China; E-Mail: tanggenfu@163.com; 7 Department of Cardiology, Peking University First Hospital, Beijing 100034, China; E-Mail: huoyong@263.net.cn; 8 Division of General Pediatrics & Adolescent Medicine, Department of Pediatrics, Johns Hopkins University School of Medicine, Baltimore, MD 21205-2179, USA

**Keywords:** homocysteine, Chinese twins, heritability, gender difference, smoking

## Abstract

Plasma homocysteine (Hcy) is a modifiable, independent risk factor for cardiovascular disease (CVD) and is affected by both environmental and genetic factors. This study aimed to describe the gender- and age-specific distribution of Hcy concentration for 1117 subjects aged 10–66 years, a subset of a community-based rural Chinese twin cohort. In addition, we examined environmental and genetic contributions to variances in Hcy concentration by gender and age groups. We found that the distribution pattern for Hcy varied by both age and gender. Males had higher Hcy than females across all ages. Elevated Hcy was found in 43% of male adults and 13% of female adults. Moreover, nearly one fifth of children had elevated Hcy. Genetic factors could explain 52%, 36% and 69% of the variation in Hcy concentration among children, male adults and female adults, respectively. The MTHFR C677T variant was significantly associated with Hcy concentrations. Smokers with the TT genotype had the highest Hcy levels. Overall, our results indicate that elevated Hcy is prevalent in the children and adults in this rural Chinese population. The early identification of elevated Hcy will offer a window of opportunity for the primary prevention of CVD and metabolic syndrome.

## 1. Introduction

Researchers have identified over 200 risk factors for cardiovascular disease (CVD) [1]. Abnormal lipids, abnormal glucose, high blood pressure, cigarette smoking and homocysteine (Hcy) are among the most important factors [1,2]. Particularly, an elevated Hcy concentration has been considered to be a modifiable, independent risk factor for the development of cardiovascular diseases (CVDs) [3]. Moreover, elevated plasma Hcy has also been associated with components of metabolic syndrome (MetS) [4]. Many of these components are also risk factors for CVD. For instance, a metabolism study found that homocysteinylation could reduce the protective effect of high-density lipoprotein (HDL) against oxidative damage and against the toxicity of Hcy-thiolactone [5]. Additionally, a four-year prospective study identified higher homocysteine levels at baseline as a significant risk factor for the development of diabetes in women with previous gestational diabetes mellitus [6]. Hcy is a sulfhydryl amino acid, whose main downregulation pathways require folic acid and vitamins B6 and B12 [7]. Inadequate B vitamin intake, particularly of folate, can lead to elevated plasma concentrations of Hcy. In addition to nutritional factors, such as folate [3], methylenetetrahydrofolate reductase (MTHFR) is another important regulatory factor for Hcy metabolism. Furthermore, the C677T polymorphism of the MTHFR gene is associated with an increased risk of hypertension [8] and stroke [9]. The risk for stroke, in particular, can be ameliorated via folate supplementation for patients with kidney disease, particularly among patients without a history of grain fortification with folic acid, as revealed by a meta-analysis [10]. As a result, a better understanding of Hcy and its correlates can be essential for CVD prevention.

There is growing recognition that early life factors may affect later CVD risk. Human health at each developmental stage is interconnected from fetal life all the way into the senior years. Although each stage presents its own unique health situations and difficulties, each stage may influence later stages. In fact, more and more compelling evidence indicates that early life health conditions may have a profound impact on health in later life [11,12]. Taking MetS and CVD as examples, both of these diseases are more commonly related to older populations. However, increasing evidence has shown that components of MetS have been identified among children and adolescents, which might help to predict the development of this and other chronic diseases, and even mortality, in the future [13]. Furthermore, early atherosclerotic lesions have also been reported in the arteries of children and adolescents [14]. As a result, childhood appears to be an even more important time window for evaluating risk factors, such as elevated Hcy levels, of CVD [15] and MetS [16].

While Hcy has been extensively studied in adults, few studies have been conducted to characterize Hcy concentration in children and adolescents [17,18,19]. One cross-sectional study of U.S. children revealed a Hcy distribution pattern (gender, race, habit) and plasma concentration association between Hcy, folic acid and vitamins B6 and B12 [17]. This study showed a substantially lower distribution of Hcy concentrations in children compared to adults; however, a small percentage of the children had elevated Hcy concentrations. Furthermore, low plasma folic acid was also considered to be a highly possible determinant of high Hcy levels in children. In support of these findings, a Japanese cross-sectional study showed that adequate folic acid and vitamin B12 levels are associated with lower blood pressure readings among preschool children [18].

Studies in China (a region with low folate intake and without universal folic acid fortification) have found widespread B vitamin deficiency and hyperhomocysteinemia in the Chinese population [20,21]. Furthermore, a hypertensive adult study in different Chinese regions has shown a high TT genotype frequency in the MTHFR gene (about 25%) [22]. To our knowledge, no previous publication has described the distributions of plasma Hcy concentration and potential environmental and genetic correlates of Hcy in a Chinese population from childhood to adulthood, particularly among those living in rural areas.

In this study, we described the gender- and age-specific distributions of plasma Hcy concentration for 1117 rural Chinese twins aged 10 to 66 years. In addition, we examined the environmental and genetic contributions to Hcy concentration by age and gender groups.

## 2. Experimental Section

### 2.1. Study Participants

This study included data from an ongoing study of MetS in a large Chinese twin cohort. The population-based cohort of twin pairs was enrolled in Anqing, China, from 1998 to 2000 (baseline) and then resurveyed in 2005–2006 (follow-up). Twins were chosen for the baseline survey based on the following criteria: (1) older than 6 years; and (2) both twins available and willing to participate. Eligible twins were invited to a central office to complete a questionnaire interview, physical examination and oral glucose tolerance test (OGTT). After a period of six years, eligible twins who met the criteria—(1) both twins participated in the baseline survey; and (2) both twins agreed and consented to participate in the follow-up study—were invited to complete the follow-up study. The total number of subjects included in the cohort at baseline and follow-up was 3413. This study included 1117 participants, a subset of the Chinese twin cohort who had Hcy concentration measured at baseline (*n* = 407) or follow-up (*n* = 710). Of note, the baseline and follow-up subjects had no overlap. As such, this study consisted of two cross-sectional samples at two time points. The baseline and follow-up groups together consisted of 1117 subjects (657 males, 460 females), aged 10–66 years. Of the 1117 subjects, 1110 (555 twin pairs) co-twins and 7 single twins were included, and 7 co-twins were excluded because of missing data. Compared to the subjects in the main cohort, our subset of study subjects had a higher average age and higher BMI and included a higher percentage of cigarette smokers, alcohol drinkers and farmers.

### 2.2. Ethics Statement

The Chinese Twin Study protocol was approved by the Institutional Review Boards (IRBs) of Ann and Robert H. Lurie Children’s Hospital of Chicago (formerly Children’s Memorial Hospital) and the Biomedical Institute, Anhui Medical University in Hefei, China. Participants aged 18 years or older gave written informed consent; for participants younger than 18 years, written informed consent was obtained from a parent/guardian and the participant. The data repository of this cohort received IRB approval (IRB Protocol Number: 00004084) from Johns Hopkins University Bloomberg School of Public Health.

### 2.3. Epidemiological Information

At both the baseline and follow-up study, a comprehensive questionnaire was used to collect the participants’ demographic, occupational and medical history.

### 2.4. Anthropomorphic Measures

Height was measured without shoes to the nearest 0.1 cm on a portable stadiometer. Weight was measured in light indoor clothing without shoes to the nearest 0.1 kg. BMI was calculated as weight (kg)/height (m) squared. Waist circumference was measured as the minimum circumference between the inferior margin of the ribcage and the crest of the ilium.

### 2.5. Blood Pressure Measurements and Definition of High Blood Pressure

Seated blood pressure measurements were obtained by trained research staff after subjects had been seated for 10 min using a mercury manometer (Hongfu Technology, Xian, China) and using the standard method of calibration and appropriately-sized cuffs. Triplicate measurements on the same arm were taken with at least 2 min between readings. Each subjects’ systolic and diastolic blood pressures were calculated as the mean of the three independent measures. Elevated blood pressure was defined as systolic blood pressure (SBP) ≥130 mmHg and/or diastolic blood pressure (DBP) ≥85 mmHg or physician diagnosed hypertension.

### 2.6. Laboratory Assay

Venous blood samples were drawn from 8:00 am to 10:00 am after a 14-h fast and collected in ethylenediaminetetraacetic acid (EDTA) tubes (Becton Dickinson Medical Devices, Shanghai, China). The samples were then centrifuged at 3000 r/min for 10 min to obtain the plasma. In our analytical center, automated biochemical analysis was used for the laboratory determinations. Concentrations of HDL-C and triglycerides (TG) were determined using enzymatic colorimetric assays (Roche Diagnostics, Shanghai, China). Plasma glucose and plasma total Hcy were measured using a Hitachi 7020 Automatic Analyzer (Hitachi, Tokyo, Japan). The MTHFR C677T genotype was determined by the Taqman assay designed and manufactured by Applied Biosystems (Applied Biosystems China, Beijing, China). The intra-assay and inter-assay coefficients of variation were <5% for all assays performed. The zygosity of each twin was determined using DNA fingerprint technology or microsatellite probes.

### 2.7. Statistical Methods

All analyses were conducted using SAS version 9.3 (SAS Institute, Cary, NC, USA). The baseline and follow-up distributions of plasma Hcy across age by gender were first examined graphically by using a locally-weighted nonparametric smoothing function LOESS, which is a generalized method of locally weighted scatterplot smoothing (LOWESS) (SMOOTH parameter = 0.8). The same method was also used to compare the period effect between baseline and follow-up subjects within each gender group in the same age range. Since the age range for pediatric purposes usually spans from birth up to age 18 (in some places, until completion of secondary education and until age 21 in the USA), we chose age 21 years as the cut-off for dividing the age groups, as has been done in previous studies [23,24]. The whole data (baseline and follow-up combined) was divided into a <21 age group and a ≥21 age group. Kernel density curves were applied for comparing gender differences in Hcy distribution within these two groups.

Multiple linear regression models were used to examine period effect, gender, alcohol status and smoking status as independent variables in relation to Hcy. All regression models were adjusted for period effect (transformed into binary variables: baseline and follow-up), age, gender, alcohol status, smoking status, education (transformed into binary variables: <middle school level and middle school and higher) and occupation (transformed into binary variables: farmer and non-farmer). Generalized estimating equations (GEE) were applied to all regression models to adjust for intra-twin pair correlation. A total of 406 subjects had MTHFR genotype data and, thus, were included in a sub-analysis. Specifically, the Wilcoxon rank sum test was applied to test the differences in median Hcy concentration between different genotypes and between smokers and nonsmokers.

The International Diabetes Federation (IDF) consensus worldwide definition of metabolic syndrome [25] is a person with central obesity (for Chinese, waist circumference ≥ 90 cm in men or ≥80 cm in women) plus two or more of the following components: SBP ≥ 130 mmHg or DBP ≥ 85 mmHg, TG ≥ 1.69 mmol/L, HDL-C < 1.03 mmol/L in men or 1.29 mmol/L in women and fasting blood glucose ≥ 5.6 mmol/L. We used these criteria for defining abnormal concentrations of these variables, as shown in the participant characteristic table (Table 1).

Classical twin modeling approaches are typically performed as a comparison between the phenotypic correlation within monozygotic (MZ) twins and also within dizygotic (DZ) twins. In our model, we assumed heritability to be the proportion of the phenotypic variance explained by additive genes. Other sources of variation assumed in the model include common or shared environment and unique environmental factors. This approach used a maximum-likelihood variance component method implemented in the statistical package, Mx [26]. Script was downloaded from the GenomEUtwin Mx-script library (http://mxscripts.ctglab.nl/index.php?page=mx_tree). Using this script, we estimated the heritability for Hcy variation in the child, male adult and female adult groups.

**Table 1:** Characteristics of participants from the Anqing twin cohort by age and gender.

Characteristics	No. (%) *			
	Child (<21 Years)		Adult (≥21 Years)	
	Male	Female	Male	Female
Number of Participants	87	58	570	402
Alcohol Use	0 (0)	0 (0)	232 (40.7)	11 (2.7)
Cigarette Use	0 (0)	0 (0)	371 (65.1)	14 (3.5)
High Fasting Glucose Level (≥5.6 mmol/L)	16 (18.4)	13 (22.4)	144 (25.3)	101 (25.1)
High Homocysteine Level (>10 μmol/L)	20 (23.0)	9 (15.5)	245 (43.0)	45 (11.2)
Low High-Density Lipoprotein (HDL) Cholesterol Level (<1.03 mmol/L for male and <1.29 mmol/L for female)	15 (17.2)	15 (25.9)	82 (14.4)	45 (11.2)
High Triglyceride Level (≥1.69 mmol/L)	8 (9.2)	5 (8.6)	63 (11.1)	42 (10.4)
High Systolic Blood Pressure (≥130 mmHg)	8 (9.2)	5 (8.6)	63 (11.1)	43 (10.8)
High Diastolic Blood Pressure (≥85 mmHg)	3 (3.4)	5 (8.6)	52 (9.2)	42 (10.5)
Middle School And Higher Education	38 (43.7)	22 (37.9)	259 (45.4)	81 (20.1)
Farmers	5 (5.8)	0 (0)	247 (48.1)	234 (58.2)
	Mean (SD)			
	Child (<21 Years)		Adult (≥21 Years)	
	Male	Female	Male	Female
Age, year	13.1 (2.5)	12.6 (1.9)	42.0 (10.9)	39.1 (8.3)
Plasma Lipids, mmol/L				
High-Density Lipoprotein (HDL) Cholesterol	1.5 (0.4)	1.4 (0.3)	1.7 (0.5)	1.6 (0.5)
Triglycerides	0.6 (0.3)	0.8 (0.3)	1.0 (0.7)	1.0 (0.6)
Blood Pressure, mmHg				
Systolic	104.3 (9.7)	101.9 (8.4)	113.6 (15.6)	108.2 (17.0)
Diastolic	59.5 (8.6)	56.1 (7.8)	72.4 (10.8)	68.6 (11.4)
Waist Circumference	57.9 (6.0)	55.6 (4.9)	73.4 (8.1)	72.5 (7.5)
Body mass index, kg/m^2^	16.4 (2.0)	15.6 (2.1)	21.6 (2.5)	22.2 (2.7)
Median and Interquartile of Plasma Total Homocysteine, μmol/L	7.5 (6.5–9.7)	6.5 (5.4–8.2)	9.6 (7.7–11.7)	7.3 (6.0–8.6)

* Total number of participants was 1117 (145 children and 972 adults). Denominators exclude participants with missing data. The percentages were calculated within each subset of total participants.

The gender-specific distribution of plasma homocysteine level across age was described by using LOESS smoothing plots for baseline subjects and follow-up subjects.

## 3. Results

### 3.1. Characteristics of the Participants

Table 1 describes the characteristics of the participants, a subset of the Anqing twin cohort. Participant data used in this study were stratified by age and gender. This rural population had a relatively low BMI in all four age groups according to clinical standards. However, the prevalence of abnormal Hcy concentrations, SBP, DBP, TG concentrations and fasting glucose concentrations was over 10% in adult males and females (except for DBP in adult females). Moreover, the children groups also showed an over 15% prevalence of elevated Hcy concentrations and an over 18% prevalence of high fasting glucose levels.

### 3.2. Age, Gender and Period Effects

**Figure 1:**
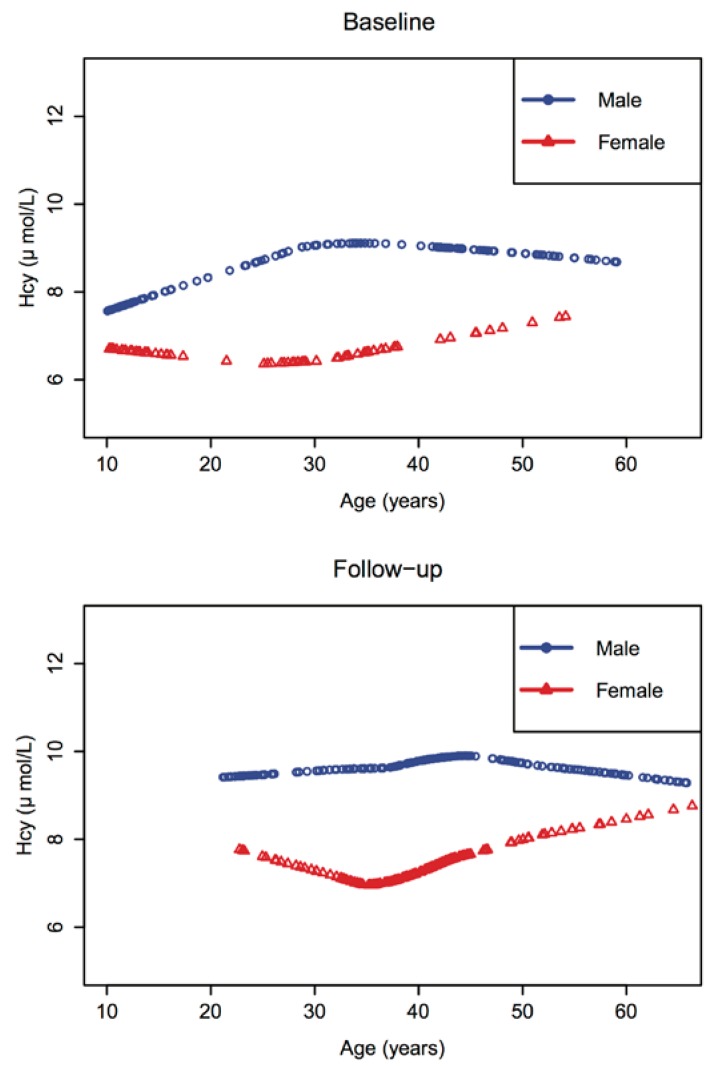
Gender-specific LOESS, a generalized method of locally weighted scatterplot smoothing (LOWESS), smoothing plots of plasma homocysteine across age.

Figure 1 displays the baseline and follow-up gender-specific locally-weighted smoothing (LOESS) plots of plasma Hcy across age. Baseline and follow-up groups shared a similar pattern of Hcy distribution. In both groups, the plasma Hcy concentrations of males were higher than those of females at every age. The largest difference between males and females occurred within the 30–40 year age period. We also compared the baseline and follow-up data within the same age range (as shown in Supplemental Figure S1). For both genders, the pattern of Hcy concentration distribution did not change much.

**Figure 2:**
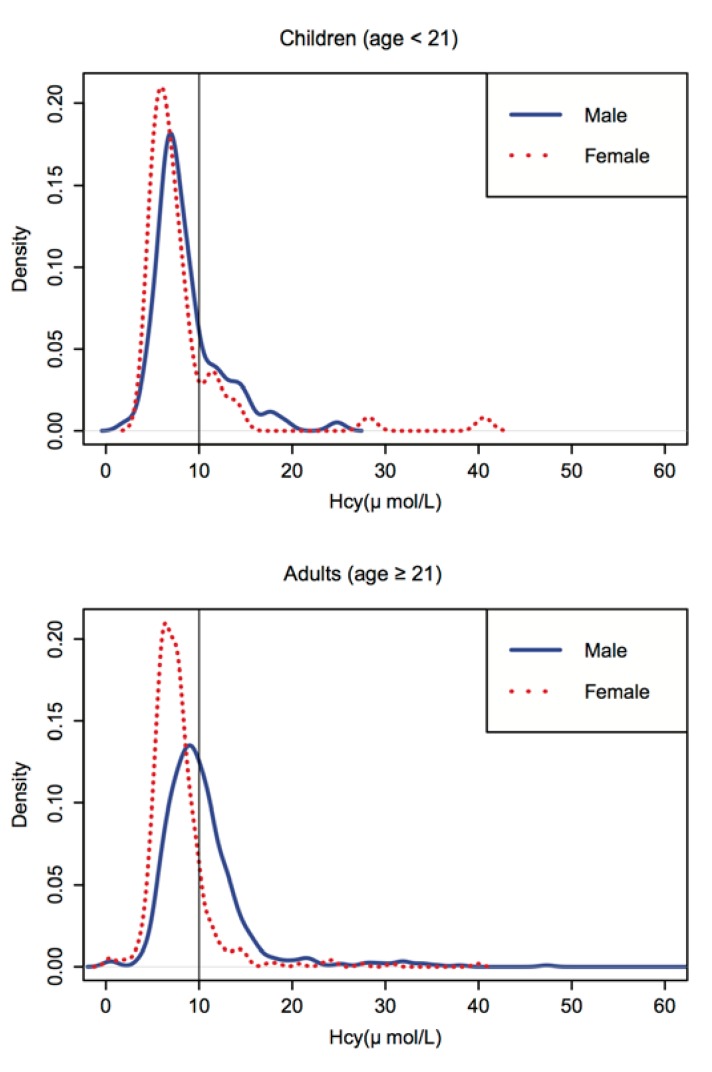
Homocysteine distribution by gender for children (age < 21) and adults (age ≥ 21) ^†^. Note: The distribution of the values was positively skewed and ranged from 0.3 to 74.7 μmol/L for the adult group and from 2.2 to 40.6 μmol/L for the child group. For both age groups, the distribution of Hcy was shifted toward higher values for males compared to females. ^†^ The distribution figure of adult homocysteine levels was trimmed at 60 for better comparison with the children group.

Figure 2 displays the children (age < 21 years) and adult (age ≥ 21 years) group gender-specific distribution curves of plasma Hcy after merging the baseline and follow-up groups. The distribution of the values was positively skewed and ranged from 0.3 to 74.7 μmol/L for the adult group, and from 2.2 to 40.6 μmol/L for the children group. For both age groups, the distribution of Hcy was shifted toward higher values for males compared to females.

### 3.3. The Effects of Genetic and Environment Factors on Hcy Concentration

The regression analyses, including the sociodemographic factors and clinical risk factors for both the adult and children groups, are shown in the Supplementary (Supplemental Table S1 and Table S2). In the adult group, an abnormal SBP was significantly associated with a higher level of Hcy (*p*-value = 0.0333). In the children group, there was a significantly slightly negative association between SBP level and Hcy level (*p*-value = 0.0048). Table 2 presents the regression analyses for all of the sociodemographic factors in relation to the plasma Hcy concentration using a GEE model excluding participants younger than age 21 years. In agreement with Figure 1 and Figure 2, gender had a significant impact on plasma Hcy concentration (β = 2.64, *p*-value < 0.0001). The regression model did not show any statistically significant period effects. Smoking was also marginally positively correlated with Hcy concentration for males (β = 0.87, *p*-value = 0.093). In addition, occupation as a farmer was also marginally positively correlated with Hcy concentration in males (β = 1.21, *p*-value = 0.063). The correlations for these sociodemographic factors and Hcy concentration were less significant within female subjects compared to male subjects.

**Table 2:** Gender-specific correlates of homocysteine levels for participants over age 21 (*n* = 972) ^†^.

	β	Standard Error	*p*-Value
Both Genders			
Follow-up (baseline as ref)	0.37	0.47	0.430
Male (female as ref)	2.64	0.52	0.000 ***
Alcohol drinking (non-drinking as ref)	−0.19	0.52	0.720
Cigarette smoking (non-smoking as ref)	0.85	0.48	0.080
Middle school or higher (lower than middle school as ref)	−0.30	0.46	0.510
Farmer (non-farmer as ref)	0.38	0.38	0.330
Male			
Follow-up (baseline as ref)	0.56	0.66	0.396
Alcohol drinking (non-drinking as ref)	−0.19	0.52	0.715
Cigarette smoking (non-smoking as ref)	0.87	0.52	0.093
Middle school or higher (lower than middle school as ref)	−0.77	0.59	0.194
Farmer (non-farmer as ref)	1.21	0.65	0.063
Female			
Follow-up (baseline as ref)	0.01	0.55	0.986
Alcohol drinking (non-drinking as ref)	0.82	0.69	0.231
Cigarette smoking (non-smoking as ref)	0.01	0.79	0.989
Middle school or higher (lower than middle school as ref)	0.60	0.68	0.377
Farmer (non-farmer as ref)	−0.44	0.34	0.196

^†^ β and *p*-value were estimated from a generalized estimating equations (GEE) model analysis of covariance that included age, sex, alcohol use, cigarette use, education and occupation. *** *p* < 0.001; ref, reference.

Moreover, we evaluated the genetic and environmental contributions to plasma Hcy concentration. The genetic factors explained 0.52 of the variance of plasma Hcy concentration in the children group. In the adult group, genetic factors explained 0.44, 0.36 and 0.69 of the variance of plasma Hcy concentration overall and in males and females, respectively (Table 3). The heritability estimation of plasma Hcy concentration was higher in females than males in the adult group. Due to the small sample size of the children group, a gender-specific heritability analysis was not possible.

**Table 3:** Heritability of homocysteine by gender and age group. MZ, monozygotic; DZ, dizygotic.

Age Group	Inter-Pair Correlation				Heritability ^†^
	MZ		DZ		
	N	R	N	R	
**<21 Year**					
Overall ^§^	33	0.80	37	0.54	0.52
**≥21 Year**					
Overall	299	0.58	173	0.36	0.44
Male	172	0.53	107	0.36	0.36
Female	127	0.55	66	0.20	0.69

Note: The heritability analysis included 542 twin pairs with zygosity data (13 twin pairs were excluded from this analysis due to missing data on zygosity). ^†^ Heritability indicates the percentage of genetic contribution to the variances in plasma homocysteine levels. ^§^ The children group did not have a large enough sample size for gender-specific heritability analysis.

**Figure 3:**
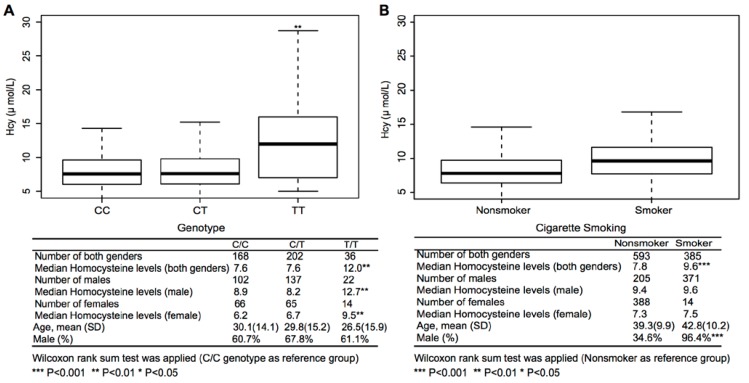
Homocysteine levels by MTHFR genotypes (**A**) and cigarette smoking profiles (**B**).

Finally, we examined a well-recognized genetic variant that may affect Hcy concentration. Figure 3 shows the boxplot of Hcy concentration by MTHFR 677C > T polymorphism and cigarette smoking profiles for both genders and the Wilcoxon rank sum test results by gender. Mean age and gender composition showed no significant differences across MTHFR genotypes (*p*-value = 0.41 and 0.33, respectively), while a significantly higher percentage of males were cigarette smokers compared to nonsmokers (96.4% *vs.* 34.6%, *p*-value < 0.001). Using the 677CC genotype as the reference, the 677TT genotype’s median Hcy concentration was statistically significantly higher than the 677CC genotype in both genders (male: *p*-value = 0.009; female: *p*-value = 0.002). Using nonsmoker as the reference, smokers did not have a significantly higher Hcy concentration than nonsmokers after we stratified the participants by gender. In addition, the smokers with the 677TT genotype showed a statistically significantly much higher risk of elevated Hcy compared to nonsmokers with the 677CC/CT genotype (*p*-value < 0.001, Table 4). No significant interaction was found between MTHFR genotype and smoking status (*p*-value = 0.365).

**Table 4:** The relative and joint effects of MTHFR genotype and cigarette use on homocysteine levels (*n* = 406) ^†^.

MTHFR C677T Genotypes	Cigarette Use	No.	β	SE	*p*-Value
CC/CT		370	reference		
TT		36	5.41	2.36	0.022 *
	no	288	reference		
	yes	118	1.35	0.67	0.043 *
CC/CT	no	262	reference		
TT	no	26	5.43	1.17	0.000 ***
CC/CT	yes	108	1.30	0.81	0.110
TT	yes	10	9.19	2.22	0.000 ***
*P* for Interaction					0.365

Note: This analysis is based on the subset data with MTHFR information. All of the samples belong to the baseline group. The sample size was 406 (261 males and 145 females). ^†^ The β and *p*-value were estimated from a GEE model analysis of covariance that included age, sex, MTHFR C677T genotype, alcohol use, cigarette use, education and occupation. *** *p* < 0.001; * *p* < 0.05.

## 4. Discussion

To our knowledge, this is the first study to reveal data on plasma Hcy concentration across the lifespan in a rural Chinese twin cohort. We demonstrated that although Hcy concentrations in children were much lower than in adults, 23% of boys and 15% of girls had elevated Hcy. We also found that the males in our study had higher Hcy concentrations than the females among both children and adults. In addition, we showed that both environmental and genetic factors affect Hcy levels. Genetic factors explained 0.52 of the variance of plasma Hcy concentration in children, while in adults, genetic factors explained 0.36 and 0.69 of the variance of plasma Hcy concentration in males and females, respectively. Finally, we examined a well-recognized gene (MTHFR) that may affect Hcy concentration and found that the 677TT genotype’s median Hcy concentration was statistically significantly higher than the 677CC genotype in both genders (male: *p*-value = 0.009; female: *p*-value = 0.002). We also found that MTHFR genotype and smoking status had a joint effect on Hcy levels. It is noteworthy that the Hcy concentration pattern for females differs from that for males within this cohort. The gender difference in Hcy concentration may be explained by both environmental and genetic factors. For instance, cigarette smoking shows a marginal association with elevated Hcy among adult males, while the association is much weaker among adult females. In addition, many studies, including our former studies, have identified the C677T polymorphism of the MTHFR gene to be significantly associated with plasma Hcy levels [27,28,29]. Consistent with these previous studies [27,28,29], we found that the MTHFR genotype contributes to elevated Hcy levels. Participants with the MTHFR 677 TT genotype have higher Hcy levels than those with the CT/CC genotype.

We went further to examine the joint effect of MTHFR genotype and smoking status. We found there to be a multiplicative joint effect between MTHFR genotype and cigarette use, since the coefficient for the joint effect (β = 9.19) was nearly equal to the product of the individual coefficients of MTHFR genotype (β = 5.41) and cigarette use (β = 1.35). As a result, subjects with the 677TT genotype appear to be more susceptible to the effect of cigarette use on Hcy levels. These findings suggest that Hcy levels may be influenced by gene × environment interactions.

In addition to smoking, we also found that the effects of occupation on plasma Hcy concentration show clear gender differences. Together, these findings indicate that males were more affected than females by detrimental environmental factors, such as smoking and occupation type. This is further supported by a differential heritability estimation for males and females (0.36 *vs.* 0.69).

The median Hcy concentration (7.2 μmol/L) for the children in our study is nearly 1.5-fold higher than the concentration found in U.S. children [17]. Meanwhile, the mean BMI of the children in our study is much lower than that of their U.S. counterparts (16.1 *vs.* 22.1). These findings may indirectly support the hypothesis regarding the importance of folic acid as a determinant of Hcy concentration starting at a young age [17,30]. In the U.S., universal folic acid fortification was initiated in 1998. In contrast, China has not yet implemented universal folic acid fortification. Moreover, there are limited sources of folate in the typical Chinese diet, and few Chinese take folic acid supplements. Therefore, it is possible that low dietary folate intake plus a lack of folic acid fortification may contribute to a relatively higher Hcy concentration, particularly among Chinese children.

The study by Osganian also suggests that multivitamin intake or supplementation with folic acid and vitamin B12 may reduce Hcy concentration in a U.S. child population [17]. Since we also found a high prevalence of elevated Hcy among children, this underscores an important opportunity for the primary prevention of CVD and MetS starting in childhood. Furthermore, Hcy-lowering via folic acid supplementation is readily available, inexpensive and safe. Given the gender difference in the contribution of environmental factors to the variance of Hcy, we suspect that folic acid supplementation may benefit males even more than females in this rural Chinese population.

One limitation of our study is the analysis of a combination of two independent cross-sectional samples. The cross-sectional nature of the analysis also limits our ability to establish causal relationships. Moreover, our study only includes a subset of the twin cohort with Hcy data (not a random sample of the rural Chinese population in Anhui province); thus, caution should be used when interpreting the results and generalizing the findings to broader populations. Looking ahead, longitudinal studies will be needed to further explore the age- and gender-related patterns of Hcy across the lifespan and temporal and dose-response relationships to CVD and MetS, as well as underlying epigenetic mechanistic studies (given that folate affects DNA methylation). Findings from these studies would be helpful for planning and evaluating future prevention methods that target Hcy regulation in different genders and age groups.

## 5. Conclusions

In summary, our results indicate that elevated Hcy was prevalent in this rural Chinese population across the lifespan and is affected by both environmental and genetic factors. Children with elevated Hcy are potentially at an increased risk for future CVD and MetS, which underscores the need for primary prevention of CVD and MetS, which should begin during early childhood.

## Supplementary Files

Supplementary File 1
