# Supplementary material for: Distribution and Determinants of Plasma Homocysteine Levels in Rural Chinese Twins across the Lifespan
Source: Nutrients. 2014 Dec 18;6(12):5900–14. doi: 10.3390/nu6125900 (PMC4277006; doi:10.3390/nu6125900)
Supplement: Supplementary File 1 [file nutrients-06-05900-s001.docx]

Supplementary Information

**Figure S1.** Gender-specific LOESS smoothing plots of plasma homocysteine across age within the same age range.


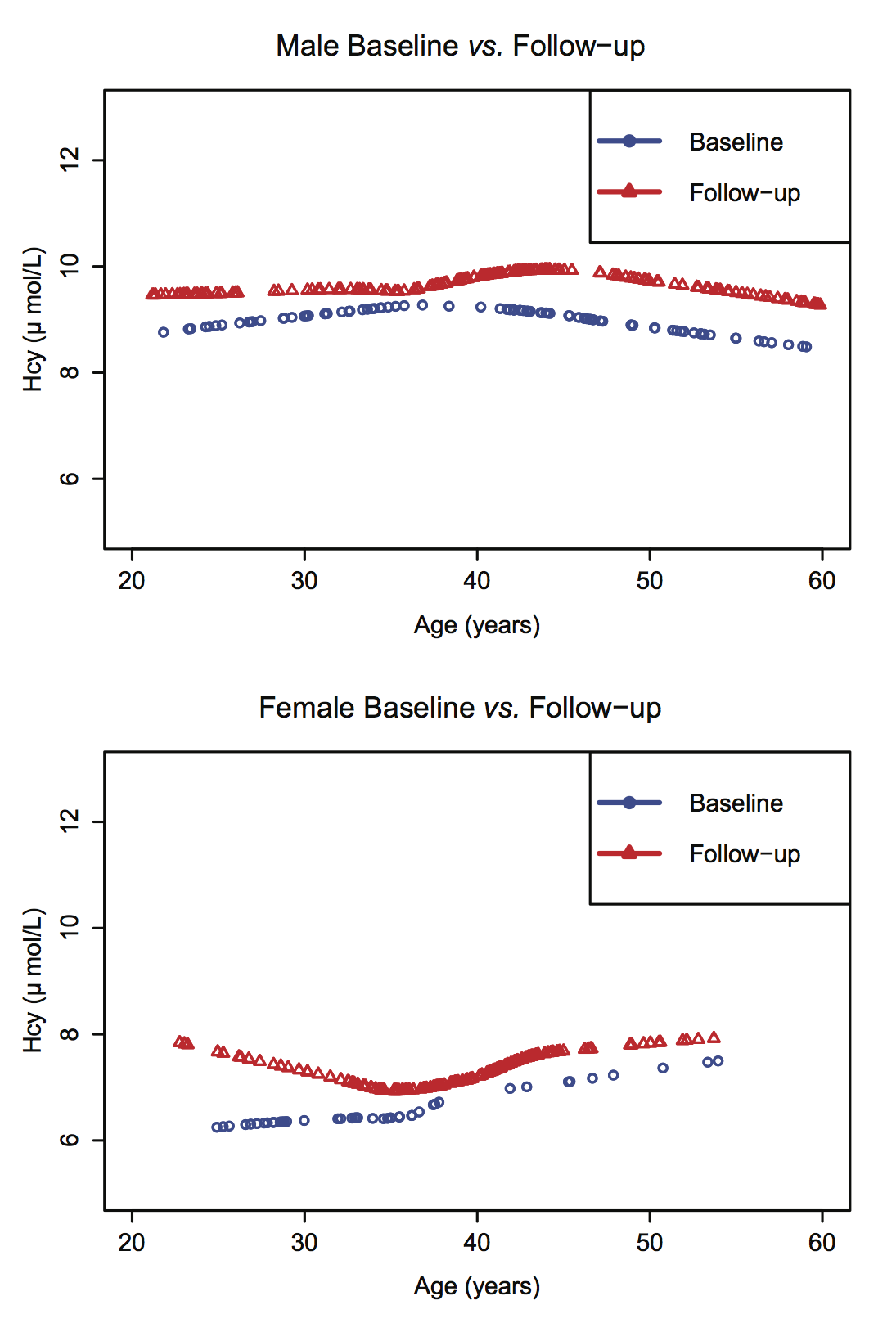


**Table S1.** Sociodemographic and clinical risk factors associated with plasma homocysteine levels in the adult group.

| **Variables** | **Adjusted Multiple Regression ^†^** | | | **Logistic Regression ^§^** | | |
| --- | --- | --- | --- | --- | --- | --- |
|  | **β** | **SE** | ***p*-value** | **OR** | **95% CI** | ***p*-value** |
| Gender |  |  |  |  |  |  |
| Female | 0.26 | 0.04 | <0.0001 | 1.38 | (1.27, 1.49) | <0.0001 |
| Male |  |  |  |  |  |  |
| Age |  |  |  |  |  |  |
| <30 |  |  |  |  |  |  |
| 30–40 | 0.02 | 0.04 | 0.566 | 0.99 | (0.89, 1.10) | 0.8741 |
| 40–50 | 0.01 | 0.04 | 0.826 | 1.02 | (0.91, 1.15) | 0.7093 |
| ≥50 | 0.08 | 0.05 | 0.149 | 0.91 | (0.80, 1.04) | 0.1535 |
| Alcohol use |  |  |  |  |  |  |
| No | 0.05 | 0.03 | 0.12 | 0.98 | (0.92, 1.05) | 0.6545 |
| Yes |  |  |  |  |  |  |
| Cigarette Use |  |  |  |  |  |  |
| No | −0.01 | 0.04 | 0.7529 | 1.00 | (0.94, 1.07) | 0.9547 |
| Yes |  |  |  |  |  |  |
| Education |  |  |  |  |  |  |
| Middle School or Higher | −0.02 | 0.05 | 0.604 | 1.00 | (0.93, 1.07) | 0.9713 |
| Lower than Middle School |  |  |  |  |  |  |
| Occupation |  |  |  |  |  |  |
| Farmer | 0.02 | 0.03 | 0.5913 | 0.99 | (0.94, 1.05) | 0.8426 |
| Non-Farmer |  |  |  |  |  |  |
| HDL-Cholesterol, mmol/L |  |  |  |  |  |  |
| Normal | −0.12 | 0.10 | 0.2047 | 0.99 | (0.87, 1.11) | 0.8171 |
| Abnormal |  |  |  |  |  |  |
| Triglycerides, mmol/L |  |  |  |  |  |  |
| Normal | 0.10 | 0.06 | 0.0686 | 1.07 | (0.98, 1.17) | 0.1516 |
| Abnormal |  |  |  |  |  |  |
| Systolic Blood Pressure, mmHg |  | |  |  | |  |
| Normal | 0.11 | 0.05 | 0.0333 | 1.07 | (0.97, 1.18) | 0.1883 |
| Abnormal |  |  |  |  |  |  |
| Diastolic blood pressure, mmHg |  |  |  |  |  |  |
| Normal | −0.03 | 0.06 | 0.6427 | 1.09 | (0.96, 1.23) | 0.1669 |
| Abnormal |  |  |  |  |  |  |
| Waist circumference, cm |  |  |  |  |  |  |
| Normal | −0.07 | 0.05 | 0.1875 | 1.02 | (0.92, 1.13) | 0.7171 |
| Abnormal |  |  |  |  |  |  |

**Table S1.** *Cont.*

| Fasting Blood Glucose, mmol/L |  |  |  |  |  |  |
| --- | --- | --- | --- | --- | --- | --- |
| Normal | 0.01 | 0.03 | 0.7116 | 1.00 | (0.94, 1.06) | 0.9979 |
| Abnormal |  |  |  |  |  |  |
| Body Mass Index, kg/m^2^ |  |  |  |  |  |  |
| 18.5–23 |  |  |  |  |  |  |
| <18.5 | −0.03 | 0.07 | 0.6528 | 1.07 | (0.94, 1.22) | 0.2775 |
| 23–25 | −0.01 | 0.03 | 0.7323 | 0.98 | (0.92, 1.05) | 0.5682 |
| ≥25 | 0.03 | 0.06 | 0.6023 | 0.93 | (0.84, 1.03) | 0.1753 |

Note: ^†^ β and *p*-values are from a GEE model analysis of covariance that included age, sex, alcohol use, cigarette use, education, occupation, HDL-C, TG, SBP, DBP, waist circumference, fasting blood glucose and BMI; ^§^ OR and the confidence interval (CI) are from a logistic GEE model analysis of covariance that included age, sex, alcohol use, cigarette use, education, occupation, HDL-C, TG, SBP, DBP, waist circumference, fasting blood glucose and BMI.

**Table S2.** Sociodemographic and clinical risk factors associated with plasma homocysteine levels in the child group.

| **Variables** | **Adjusted Multiple Regression ^†^** | | | **Logistic Regression ^§^** | | |
| --- | --- | --- | --- | --- | --- | --- |
|  | **β** | **SE** | ***p*-value** | **OR** | **95% CI** | ***p*-value** |
| Gender |  |  |  |  |  |  |
| Female | 0.09 | 0.10 | 0.3598 | 0.98 | (0.82, 1.17) | 0.8047 |
| Male |  |  |  |  |  |  |
| Age |  |  |  |  |  |  |
| <13 |  |  |  |  |  |  |
| 13–18 | 0.22 | 0.16 | 0.1685 | 1.06 | (0.79, 1.41) | 0.7071 |
| ≥18 | 0.48 | 0.21 | 0.0223 | 1.16 | (0.63, 2.14) | 0.6324 |
| Education |  |  |  |  |  |  |
| Higher than Middle School | 0.02 | 0.10 | 0.8726 | 0.95 | (0.79, 1.14) | 0.5647 |
| Middle School or Lower |  |  |  |  |  |  |
| Occupation |  |  |  |  |  |  |
| Farmer | −0.25 | 0.11 | 0.0211 | 0.72 | (0.52, 1.01) | 0.0535 |
| Non-Farmer |  |  |  |  |  |  |
| HDL-Cholesterol, mmol/L | 0.06 | 0.08 | 0.4243 | 1.05 | (0.90, 1.21) | 0.5563 |
| Triglycerides, mmol/L | −0.08 | 0.09 | 0.4176 | 0.83 | (0.68, 1.02) | 0.079 |
| Systolic Blood Pressure, mmHg | −0.01 | 0.00 | 0.0048 | 0.99 | (0.98, 1.00) | 0.0057 |
| Diastolic Blood Pressure, mmHg | 0.00 | 0.01 | 0.7177 | 1.01 | (1.00, 1.02) | 0.1406 |
| Waist Circumference, cm | 0.00 | 0.01 | 0.7754 | 1.00 | (0.99, 1.02) | 0.5537 |
| Fasting Blood Glucose, mmol/L | 0.01 | 0.04 | 0.7997 | 0.98 | (0.88, 1.09) | 0.7186 |
| Body Mass Index, kg/m^2^ | 0.02 | 0.02 | 0.3232 | 1.03 | (0.99, 1.08) | 0.137 |

Notes: ^†^ β and *p*-values are from a GEE model analysis of covariance that included age, sex, education, occupation, HDL-C, TG, SBP, DBP, waist circumference, fasting blood glucose and BMI; ^§^ ORs and confidence intervals (CI) are from a logistic GEE model analysis of covariance that included age, sex, education, occupation, HDL-C, TG, SBP, DBP, waist circumference, fasting blood glucose and BMI.
